# *Scutellaria barbata* flavonoids alleviate memory deficits and neuronal injuries induced by composited Aβ in rats

**DOI:** 10.1186/s12993-016-0118-8

**Published:** 2016-12-08

**Authors:** Xiao G. Wu, Shu S. Wang, Hong Miao, Jian J. Cheng, Shu F. Zhang, Ya Z. Shang

**Affiliations:** 1 Hebei Province Key Research Office of Traditional Chinese Medicine Against Dementia/Institute of Traditional Chinese Medicine, Chengde Medical College/Hebei Province Key Laboratory of Traditional Chinese Medicine Research and Development, Chengde, Hebei 067000 China; 2 Hebei Research Institute for Family Planning, Shijiazhuang, Hebei 050000 China

**Keywords:** *Scutellaria barbata* flavonoids, Aβ 25-35, AlCl3, RHTGF-β1, Memory, Neuronal injuries

## Abstract

**Background:**

The aim of the present study was to investigate the effects of *Scutellaria barbata* flavonoids (SBF) on memory impairment and neuronal injury induced by amyloid beta protein 25–35 in combination with aluminum trichloride (AlCl3) and recombinant human transforming growth factor-β1 (RHTGF-β1) (composited Aβ) in rats.

**Methods:**

The composited Aβ-treated model of Alzheimer’s disease (AD)-like memory impairment and neuronal injury was established in male rats by right intracerebroventricular injection of composited Aβ, and the effects of SBF were assessed using this rat model. Spatial learning and memory of rats were assessed in the Morris water maze, and neuronal injury was assessed by light and electron microscopy with hematoxylin-eosin or uranyl acetate and lead nitrate-sodium citrate staining, respectively.

**Results:**

In the Morris water maze, memory impairment was observed in 94.7% of the composited Aβ-treated rats. The composited Aβ-treated rats took longer than sham-operated rats to find the hidden platform during position navigation and reversal learning trials. They also spent less time swimming in the target quadrant in the probe trial. Optical and electron microscopic observations showed significant neuropathological changes including neuron loss or pyknosis in hippocampus, typical colliquative necrosis in cerebral cortex, mitochondrial swelling and cristae fragmentation and a large number of lipofuscin deposits in the cytoplasm. Treatment with SBF (35–140 mg/kg) reduced the memory impairment and neuronal injury induced by composited Aβ.

**Conclusion:**

SBF-mediated improvement of composited Aβ-induced memory impairment and neuronal injury in rats provides an appropriate rationale for evaluating SBF as a promising agent for treatment of AD.

**Electronic supplementary material:**

The online version of this article (doi:10.1186/s12993-016-0118-8) contains supplementary material, which is available to authorized users.

## Background

Alzheimer’s disease (AD) is a chronic and progressive neurodegenerative disease in the elderly, and it is accompanied by gradual memory loss. In general, atrophy of the nervous system, loss of neurons and synapses, as well as disorders of subcellular structure and function are closely associated with the occurrence and development of AD [1, 2]. In particular, extracellular senile plaques (SP), which are primarily composed of aggregated beta-amyloid (Aβ), and intracellular neurofibrillary tangles (NFT), which are composed of insoluble aggregates of hyperphosphorylated tau protein in the brain, are considered the most important histopathogenic traits in AD. Multiple neurotoxic events in the brain, such as Aβ aggregation, tau protein hyperphosphorylation, disruption of calcium homeostasis, and production of reactive oxygen species, have been shown to occur when animals were intraventricularly injected with Aβ [3]. The deposited Aβ may result in massive SP and NFT formation, and the combined effects of deposited Aβ and hyperphosphorylated tau protein exacerbate neurotoxicity and advance dementia [4]. An animal model of AD was established using Aβ25–35 in combination with aluminum trichloride (AlCl3) and recombinant human transforming growth factor-β1 (RHTGF-β1) injected into the lateral cerebral ventricle (composited Aβ-treated rat). This model provides a comprehensive simulation of human histopathogenic traits [5]. Aluminum can prevent conversion of sedimentary Aβ into soluble Aβ, and RHTGF-β1 can enhance sedimentary Aβ formation and accelerate occurrence of AD [6]. Thus, several composited Aβ-induced neuronal dysfunctions are relevant to AD, and an intervention that can decrease composited Aβ-mediated neuronal injury may be useful in the treatment of AD.

*Scutellaria barbata* flavonoids (SBF), which are isolated from the aerial parts of *S*. *barbata* D. Don, have been shown to alleviate fever, inflammation, peroxidation, as well as improve memory deficits and neuroendocrine and abnormal free radical changes in ovariectomized rats [7–9]. However, the effects of SBF on impaired learning and memory and neuronal damage induced by composited Aβ in rats has not been reported. In the present study, the effects of SBF were assessed using a composited Aβ-treated rat model of AD-like memory impairment and brain injury, which was established by intracerebroventricular injection of Aβ25–35 in combination with AlCl3 and RHTGF-β1.

## Materials

### Animals

Four-month-old male Sprague–Dawley rats were purchased from the Experimental Animal Center of Hebei Medical University (Clean grade, Certification No. scxk (Ji) 2010-1-003). Rats were housed in groups of four or five per cage with free access to food and water under controlled laboratory conditions with a 12-h light–dark cycle and an ambient temperature of 22–24 °C. Before the operation, the rats were allowed to acclimatize to the laboratory environment for 1 week. All animal procedures were carried out in accordance with the Regulations of Experimental Animal Administration issued by the State Committee of Science and Technology of China on Oct. 31, 1988 [10]. All efforts were made to minimize the animal number and their discomfort.

### Drug and reagents

SBF was prepared by the Phytochemistry Laboratory, Institute of Traditional Chinese Medicine, Chengde Medical College, Chengde City, China. One kg of dried aerial parts of *S*. *barbata* D. Don was boiled twice for 1 h with 80% alcohol, and the extract was filtered with filter paper. The filtration was performed, and the extract was evaporated under reduced pressure until no alcohol remained. The concentrated solution was adjusted to pH 2 by adding 1 N HCl and was maintained at room temperature for 24 h until the sediment completely formed. The sediment was SBF, and the flavonoid was not less than 85%. Scutellarein was the major ingredient as shown by high performance liquid chromatography assay [11]. Aβ25-35, AlCl3 and RHTGF-β1 were purchased from Shanghai Qiangyao Bioabiotechnology Co., Ltd (Shanghai, China), Tianjin Beichen Reagent Company Inc (Tianjin, China) and Prospect Biosystems (Newark, NJ, USA), respectively. Other reagents were AR grade and were supplied by commercial sources.

## Methods

### Surgical procedure

One hundred male Sprague–Dawley rats (300–350 g, 4 months of age) were used in the experiments. Eighty rats were microinjected with composited Aβ into the right lateral cerebral ventricle and designated as composited Aβ-treated rats. Twenty rats were subjected to a sham operation. The rats were anaesthetized with 10% chloral hydrate (300 mg/kg, intraperitoneal) and restrained in a brain stereotaxic apparatus (RWD, Shenzhen, China). On the first day of the operation, as shown in Additional files 1 and 2, 1 μL of RHTGF-β1 (10 ng) was microinjected into the lateral cerebral ventricle area [posterior (P): 1.0 mm to the bregma, lateral (L): 1.4 mm to the midline, and ventral (V): 4.6 mm to the skull]. A catheter was inserted into the lateral cerebral ventricle area [posterior (P): 1.2 mm to the bregma, lateral (L): 2.0 mm to the midline, and ventral (V): 4.6 mm to the skull] [12]. On the second day of operation, 4 μg (1 μL) Aβ25–35 and 3 μL AlCl3 (1%) were microinjected daily for 14 days in the morning and 5 days in the afternoon, respectively. The sham-operated group was subjected to the same operation and received a saline microinjection. Seventy-six composited Aβ-treated rats survived, the success rate of the operation was 95%. Eighteen sham-operated rats survived, the success rate of the operation was 90%.

### Experimental design

**Fig. 1:**
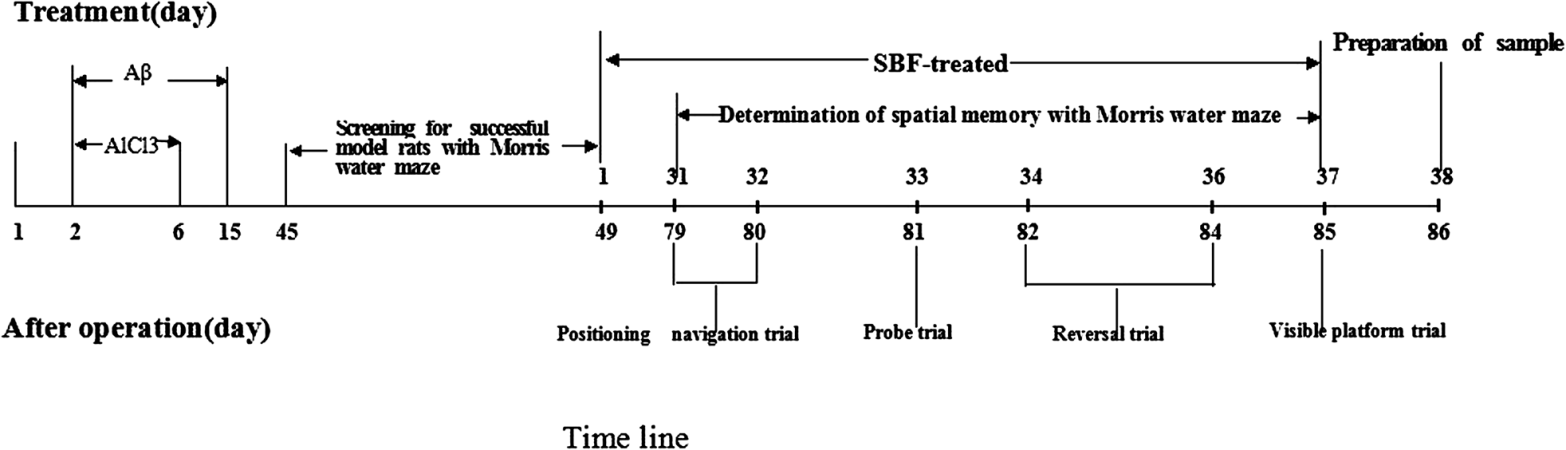
The timeline of experimental design

The entire experiment took 86 days and Fig. 1 showed the timeline of experimental design. All rats were allowed to recover for 45 days after the operation. The Morris water maze was used to screen rats for learning deficits and to assess their spatial memory. The rats underwent 4 consecutive days of water maze training with 2 trials per day. Composited Aβ-treated rats that displayed a learning deficit on day 4 of training in the Morris Water Maze were randomly divided into 4 groups: composited Aβ-treated group or 3 drug-treated groups (3 doses). Rats in the drug-treated groups were administered 35, 70 and 140 mg/kg (oral) of SBF daily for 38 days. The sham-operated rats were given saline. The rats’ spatial memory was tested in the Morris water maze over 7 consecutive days, from day 31 to day 37 of SBF administration (namely, day 79 to day 85 after the operation). The medication lasted throughout the Morris water maze test period. All the rats were killed by decapitation 60 min after the last administration of SBF or saline on day 38 of administration.

### Screening for successful model rats and assessment of behavior in the Morris Water Maze

The Morris water maze was used to assess learning and memory and screen for successful model rats [13]. The Morris water maze was a stainless steel circular pool with a diameter of 120 cm and a depth of 50 cm. It was purchased from the Institute of Materia Medica, Chinese Academy of Medical Science and Peking Union Medical College (Beijing, China). When the water maze test was performed, the pool water was blackened with several drops of ink. The water depth was 31.5 cm, and the temperature was maintained at 23 ± 1 °C. A circular transparent plexiglass platform was set 1.5 cm below the water surface. Each spatial signal around the maze was invariable during all water maze tests. For descriptive data collection, the pool was subdivided into four equal quadrants formed by imaginary lines. The hidden platform was placed in the first quadrant (Q1). All swimming behaviors (measured by latency or trajectory) of rats were captured by a video camera linked to computer-based graphics analytic software (Institute of Materia Medica, Chinese Academy of Medical Science and Peking Union Medical College).

### Screening for successful memory impairment of composited Aβ-treated rats

On day 45 after the operation, all rats underwent four consecutive days of Morris water maze training to screen for memory impairment (screening for successful composited Aβ-treated rats) (Fig. 1). The screening ratio (SR) was calculated from the average latency to find the hidden platform on day 4 of water maze training for each composited Aβ-treated rat and sham-operated rats. The average latency to find the hidden platform on day 4 of water maze training for each composited Aβ-treated rat was “A”, and the average latency of sham-operated rats was “B”. Then, SR = (A − B)/B. When SR was larger than 0.2 for a composited Aβ-treated rat, it was considered a successful composited Aβ-treated rat with impaired memory [14].

### Determination of spatial memory

Spatial memory was assessed for seven consecutive days with two trials per day using the Morris water maze. The time spent finding the hidden platform was recorded, and an average value was calculated from the data of two trials to determine intraday memory performance. The water maze test procedure was designed such that the rats were allowed to swim and search for the hidden platform within 60 s. If a rat missed the hidden platform within 60 s, the experimenter then placed the rat on the platform. When a rat reached the hidden platform (independently or assisted), the rat was allowed to remain there for 20 s, and then the rat was removed from the pool. Each rat was allowed a 10 s recovery time between the two trials. Memory measurement was divided into four parts: 2 days of positioning navigation trial, 1 day of probe trial, 3 days of reversal trial, and finally 1 day of visible platform trial [15].

### Positioning navigation trial

The positioning navigation trial was used to evaluate memory acquisition on days 1 and 2 in the Morris water maze. This was performed on days 31 and 32 after initiation of treatment with SBF, which corresponded to days 79 and 80 after the operation (Fig. 1). The location of the hidden platform was the same as during screening of the composited Aβ-treated rats (Q1). The average value of latency over 2 trials was taken as the intraday memory acquisition score.

### Probe trial

The probe trial was used to evaluate memory retention on day 3 of the Morris water maze test, which was conducted on day 33 after initiation of treatment with SBF and day 81 after the operation (Fig. 1). The platform was removed from the pool, and the rats were allowed to swim 60 s and search for the target quadrant (Q1) where the platform was located during the positioning navigation trial. Swimming time in the target quadrant (Q1) was recorded for 60 s and taken as the memory retention score.

### Reversal trial

The reversal trial was used to evaluate re-learning for three consecutive days on days 4, 5, and 6 of the Morris water maze test, which corresponded to day 34, 35 and 36 of SBF treatment and day 82, 83 and 84 after the operation (Fig. 1). The platform was placed on the opposite side of the target quadrant (Q3). The average latency over two trials was taken as the rats’ intraday re-learning achievement.

### Visible platform trial

The visible platform trial was used to evaluate swimming speed on day 7 of the Morris water maze test. The aim was to exclude the influence of motivational or sensorimotor factors upon learning and memory performance. All rats were subjected to a 1 day visible platform trial on day 37 after initiation of SBF treatment, which corresponded to day 85 after the operation (Fig. 1). The platform was elevated 2 cm above the water surface. The swimming speed of all rats in the pool was recorded.

### Detection of neuronal injury

Under ether anesthesia, the rats were killed by decapitation 60 min after the last administration of SBF or saline on day 38 of treatment (Fig. 1). For three rats from each group, the right hemisphere was gently separated on ice and then routinely processed as previously described [16]. Coronal sections, approximately 4 µm-thick, were cut and stained with hematoxylin-eosin (HE). Stained neurons were visualized and photographed at a magnification of 4× or 400× using an Olympus VANOX microscope from Olympus Optical Co. Ltd. (Tokyo, Japan). An investigator blinded to the experimental design counted neurons per 0.125 mm in the CA1 region of the hippocampus and per 0.0352 mm^2^ of the cerebral cortex at 400×. Three subfields of the hippocampal CA1 region and cerebral cortex were selected from each rat brain. The average number of normal neurons was determined in each group at a magnification of 400×. Neurons were identified as normal if they appeared undamaged with round or oval cell bodies, which distinguished them from glial cells. In addition, hippocampi of the left hemisphere were double-fixed with 2.5% glutaraldehyde and 1% osmic acid and then sectioned with an ultramicrotome. The sections were placed on a 200-mesh copper grid and stained with uranyl acetate and lead nitrate-sodium citrate as described previously [17]. The ultrastructure of cells was observed with a JEOL 100CX II transmission electron microscope and photographed at a magnification of 10,000–35,000×.

### Statistical analysis

Data are presented as mean ± SEM. Statistical analysis was performed using a SAS/STAT Microsoft package obtained from SAS, USA. Two-way analysis of variance (ANOVA) with repeated measures was used to analyze group differences in latency to reach the platform in the Morris water maze test, and one-way ANOVA followed by Duncan’s multiple-range test was used to analyze group differences in the probe trial and the number of neurons. Differences with *P* values <0.05 were considered statistically significant.

## Results

### Screening AD model rats using the Morris water maze test

**Fig. 2:**
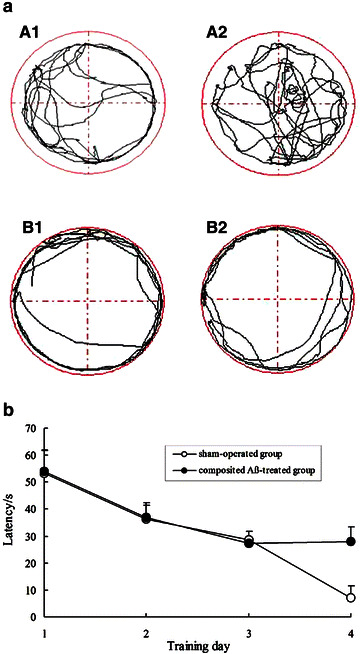
Screening for memory impairment of rats using the Morris water maze. **a** The adaptive swimming trajectory of rats in the Morris water maze. *A1*–*A2* Sham-operated rats; *B1*–*B2* composited Aβ-treated rats. **b** Mean latency to find the hidden platform for 4 consecutive days of screening trials in the Morris water maze for sham-operated and composited Aβ-treated rats

In recording adaptive swimming, we found that the sham-operated rats always swam freely, and the composited Aβ-treated rats always swam around the pool perimeter (Fig. 2a). Over the 4 days of testing model rats in the Morris water maze, the time to find the hidden platform (latency) progressively declined in all animals. When the screening ratio (SR), which was based on the latency to find the hidden platform on day 4 for composited Aβ-treated and sham-operated rats, was more than 0.2, this animal was considered as a successful model rat. The percentage of successful model rats was 94.7% (Fig. 2b).

### Effect of SBF on rat memory acquisition in the Morris water maze test

**Fig. 3:**
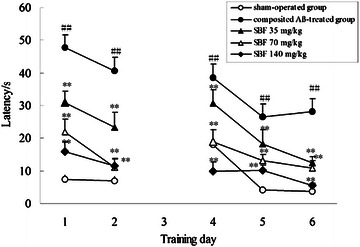
Effects of SBF on memory acquisition and re-learning impairment induced by composited Aβ in rats. The positioning navigation trial was used to evaluate memory acquisition by 2 consecutive days swimming achievement on day 1 and 2 in the Morris water maze test. These were performed on day 31 and 32 after initiation of treatment with SBF, which corresponded to day 79 and 80 after the operation. The reversal trial was used to evaluate memory re-learning of rats by 3 consecutive days swimming score on day 4, 5, and 6 in the Morris water maze test, which corresponded to day 34, 35 and 36 of SBF treatment, namely on day 82, 83 and 84 after the operation. The *line graph plots* showed the mean latency to find the hidden platform for each group on day 1, 2, 4, 5, and 6 in the Morris water maze test. Data were analyzed by two-way ANOVA (day × group) with repeated measures. Mean ± SEM. n = 6. ^##^ *P* < 0.01, vs. sham-operated group. **P < 0.01, vs. composited Aβ-treated group

The positioning navigation trial was used to evaluate rat memory acquisition on day 1 and 2 of the Morris water maze test. During the 2 days memory acquisition trial, the latency to find the hidden platform progressively declined in all rats. However, as shown in Fig. 3, the latency of the composited Aβ-treated group was 540% and 454% [*F* (1, 6) = 187.37, *P* < 0.01] greater than that of the sham-operated group on days 1 and 2, respectively. The prolonged latency of the composited Aβ-treated group was significantly shortened by treatment with SBF at doses of 35 mg/kg [*F* (1, 6) = 5.71, *P* < 0.05], 70 mg/kg [*F* (1, 6) = 17.51, *P* < 0.01], and 140 mg/kg [*F* (1, 6) = 79.67, *P* < 0.01].

### Effect of SBF on rat memory retention in the Morris water maze test

**Fig. 4:**
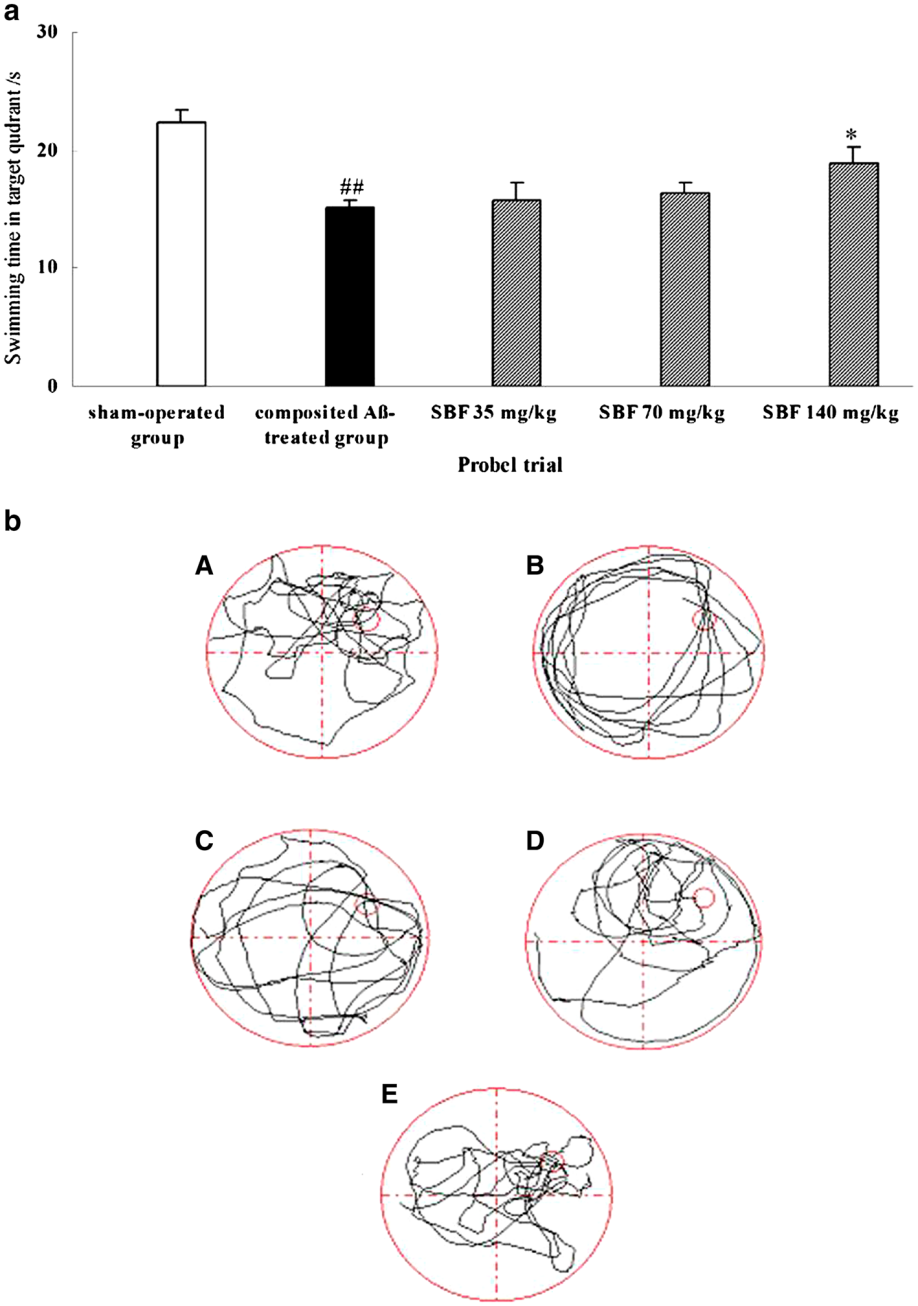
Effects of SBF on memory retention impairment induced by composited Aβ in rats. The probe trial was used to evaluate memory retention of rats by 1 day swimming achievement on day 3 in the Morris water maze test, which was conducted on day 33 initiation of SBF treated, namely on day 81 after the operation. **a** Time spent swimming in the target quadrant within 60 s in the probe trial (no platform). Data were analyzed by one-way ANOVA with the multiple-range test. Mean ± SEM. n = 6. ^##^ *P* < 0.01, vs. sham-operated group. *P < 0.05 vs. composited Aβ-treated group. **b** Typical swimming-tracking paths of rats in probe trial. *A* Sham-operated group, *B* Composited Aβ-treated group, *C* SBF 35 mg/kg group, *D* SBF 70 mg/kg group, *E* SBF 140 mg/kg group

The probe trial was used to evaluate rat memory retention on day 3 of the Morris water maze test. As shown in Fig. 4a and b, the time that composited Aβ-treated rats swam in the target quadrant (Q1) decreased by 32.14% within 60 s compared with sham control rats [*F* (1, 6) = 7.16, *P* < 0.05]. The reduced swimming time of the composited Aβ-treated group was differently attenuated by 3 doses of SBF, which increased swimming time 4.63% in response to 35 mg/kg SBF, 8.40% in response to 70 mg/kg SBF, and 25.26% in response to 140 mg/kg SBF [*F* (1, 6) = 3.82, *P* < 0.05].

### Effect of SBF on rat memory re-learning in the Morris water maze test

The reversal trial was used to evaluate rat memory re-learning on days 4, 5, and 6 of the Morris water maze test. Figure 3 shows that the composited Aβ-treated rats took 113, 521, and 652% longer to find the hidden platform than the sham control rats [*F* (1, 6) = 26.55, *P* < 0.01]. It is interesting that on days 4, 5, and 6 of the Morris water maze test, the 3 doses of SBF differentially shortened the longer latencies, which decreased 20.43, 31.24, and 55.53% in response to 35 mg/kg SBF [*F* (1, 6) = 7.23, *P* < 0.05], 51.77, 50.11, and 61.08% in response to 70 mg/kg SBF [*F* (1, 6) = 17.51, *P* < 0.01], and 74.04, 61.5, and 80.51% in response to 140 mg/kg SBF [*F* (1, 6) = 79.67, *P* < 0.01].

### Effect of SBF on rat swimming speed in the Morris water maze test

The visible platform trial was used to evaluate rat swimming speed on day 7 of training in the Morris water maze test. The times spent finding the visible platform for rats in each group were not significantly different [*F* (4, 30) = 0.79, *P* > 0.05]. Therefore, individual differences in rat swimming speed could be excluded, which indicated that motivation and motor skills were essentially intact.

### Effect of SBF on rat neuronal injuries induced by composited Aβ-treatment

**Fig. 5:**
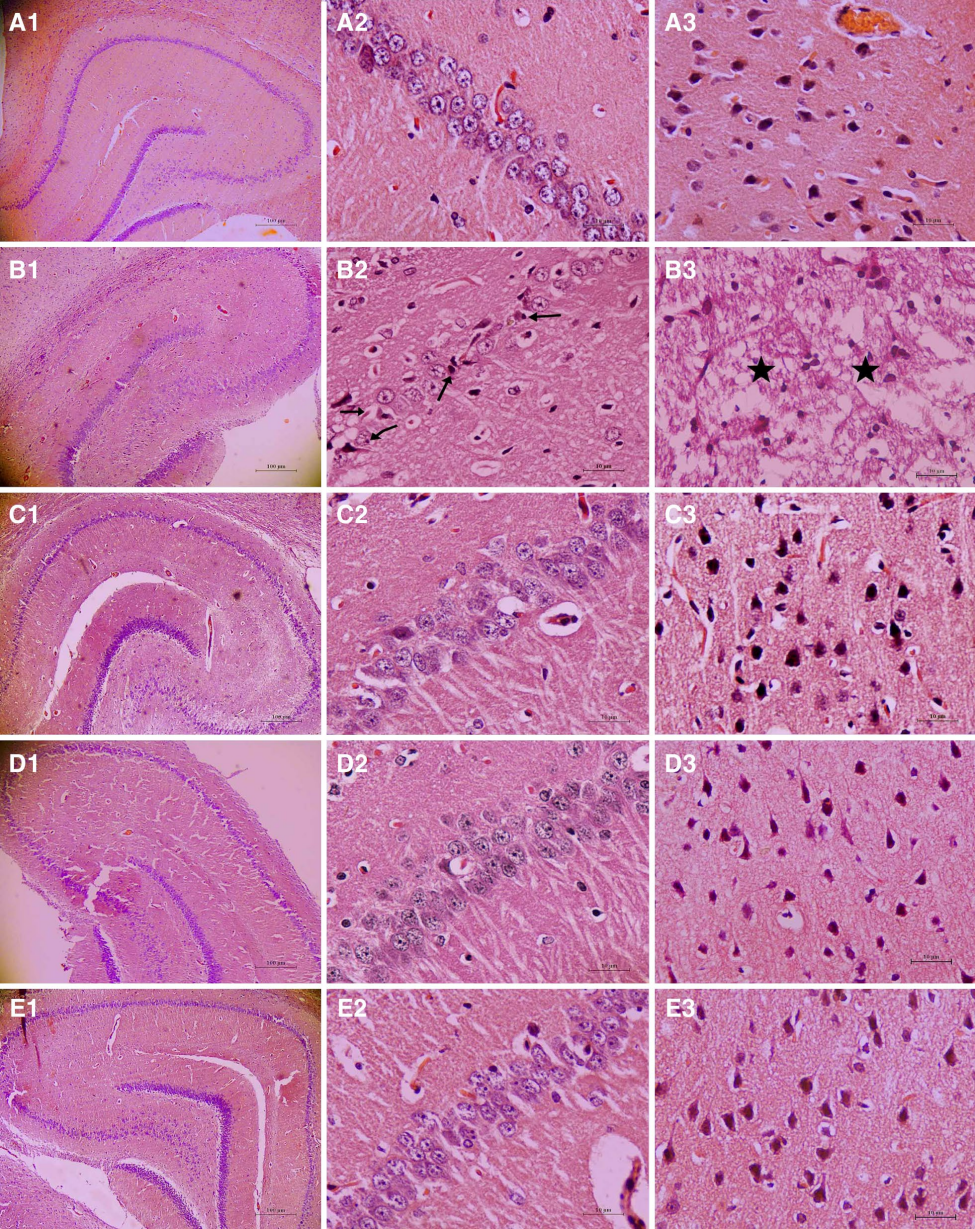
Effects of SBF on pathological changes in the hippocampus and cerebral cortex induced by composited Aβ in rats. Representative images of hippocampal and cerebral cortical neurons stained with HE. *A1*–*E1* Hippocampus ×40; *A2*–*E2* Hippocampus CA1 ×400; *A3*–*E3* Cerebral cortex ×400. *A1*–*A3* Sham-operated group; *B1*–*B3* Composited Aβ-treated group; showing loss of neurons, neurofibrillary degeneration (→), neuronophagia (←), nuclear pyknosis (↗), nuclear margination (↙), colliquative necrosis (★)with disrupted cellular membranes. Nuclei were fragmented, and large numbers of inflammatory cells infiltrated regions of the cerebral cortex in composited Aβ-treated rats. *C1*–*C3* SBF 35 mg/kg group; *D1*–*D3* SBF 70 mg/kg group; *E1*–*E3* SBF 140 mg/kg group. *Scale bar* 10 or 100 µm

Three rats from each group were decapitated 60 min after the last administration of SBF or saline on day 38 of drug treatment. In several composited Aβ-treated rats, visual inspection revealed a yellow surface, and a thin or collapsed cerebral cortex. Optical microscopy of HE stained brains from the composited Aβ-treated group showed marked pathological changes in neurons of the hippocampus and cerebral cortex, such as neurofibrillary degeneration, neuronophagia, nuclear pyknosis, and nuclear margination (Fig. 5aB1, B2), as compared with the sham-operated group (Fig. 5aA1, A2, A3). In addition, neurons in part of the cerebral cortex of composited Aβ-treated rats showed typical colliquative necrosis, which was characterized by disrupted cell membranes, fragmented nuclei, and extensive infiltration of inflammatory cells in the necrotic region (Fig. 5aB3). However, in composited Aβ-treated rats that had been treated with SBF for 38 d, neuronal injuries in the hippocampus and cerebral cortex were markedly attenuated in a dose-dependent manner (Fig. 5aC1–E1, C2–E2, C3–E3).

**Fig. 6:**
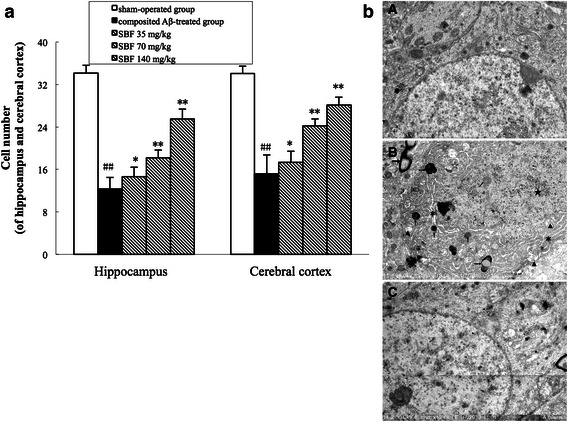
**a** Numbers of neurons in the hippocampus and cerebral cortex, which were counted under a light microscope (×400). Each volume represents mean ± SEM from nine visual fields of three independent samples (n = 3). ^##^ *P* < 0.01, vs. sham control. *P < 0.05, **P < 0.01, vs. composited Aβ-treated. **b** Subcellular structure of hippocampal neurons assessed by electron microscopic observation. *A* Sham-operated group ×12,000, *scale bar* 4 µm; *B* Composited Aβ-treated group; showing mitochondrial swelling, cristae fragmentation, and increased electron density (▲), rough endoplasmic reticulum dilation (_*_), polyribosome and polymicrotubule depolymerization, secondary lysosome (↑) production, a large number of lipofuscin (→) deposits in the cytoplasm, rough and curved nuclear membranes, condensed and denatured euchromatin (★), loose or attenuated myelin sheath layers, internal axon and fiber degeneration, and almost normal golgiosomes. ×10,000, *scale bar* 5 µm; *C* SBF 140 mg/kg group, ×12,000, *scale bar* 4 µm

In addition to pathological changes, the number of neurons was significantly reduced in the brains of composited Aβ-treated rats, as compared with those of the sham-operated group. The neuron count was 63.86 ± 4.35% (*P* < 0.01) lower than that of the sham-operated group in 0.125 mm sections of the hippocampal CA1 area and 55.46 ± 5.48% (*P* < 0.01) lower in 0.0352 mm^2^ sections of the cerebral cortex (Fig. 6a). It is noteworthy that the decreased neuron count in composited Aβ-treated rats was dramatically reversed by treatment with SBF for 38 days. The number of neurons was increased 18.98% by 35 mg/kg (*P* < 0.05), 47.36% by 70 mg/kg (*P* < 0.01), and by 140 mg/kg 106.81% (*P* < 0.01) in the hippocampus CA1 subfield and 14.24% by 35 mg/kg (*P* < 0.05), 59.33% by 70 mg/kg (*P* < 0.01), and 85.63% by 140 mg/kg (*P* < 0.01) in the cerebral cortex subfield (Fig. 6a).

The ultrastructure of neurons was examined with electron microscopy. Compared with the sham-operated group (Fig. 6cA), neurons in the composited Aβ-treated group were severely damaged, showing mitochondrial swelling and cristae fragmentation, increased mitochondrial electron density, dilation of the rough endoplasmic reticulum, depolymerization of polyribosomes and polymicrotubules, smaller postsynaptic density (PSD), production of secondary lysosomes, and a large number of lipofuscin deposits in the cytoplasm. The nuclear membrane appeared rough and sunken, euchromatin was condensed and denatured, myelin sheath layers were loose or attenuated, and internal axons and fibers were degenerated (Fig. 6cB). However, 140 mg/kg SBF administered for 38 days dramatically attenuated these neuronal pathological changes induced by composited Aβ, and damage to neuronal subcellular structure was reduced (Fig. 6bC).

## Discussion

It is well known that the loss of learning and memory is the major clinical symptom in AD patients [18]. In the present study, the Morris water maze was used to assess memory impairment in the AD-like model rat. We found that the percentage of successful model rats was 94.70%, which indicated that the established method for screening model rats injected with Aβ25–35 in combination with AlCl3 and RHTGF-β1 was credible. These successful model rats were used to measure the effects of SBF. On day 1 and 2 of the positioning navigation trial, the rats in the composited Aβ-treated group took longer to find the hidden platform than the sham-operated group, which demonstrated impaired spatial memory acquisition in the composited Aβ-treated rats. On day 3 of the probe trial, the rats in the composited Aβ-treated group spent less time swimming in the target quadrant, which indicated decreased memory retention in composited Aβ-treated rats. On day 4, 5, and 6 of the memory re-learning trial, the rats in the composited Aβ-treated group required more time to find the hidden platform compared with rats in the sham-operated group. This result suggests that the composited Aβ can impair memory re-learning. However, when the composited Aβ-treated rats were treated with 35, 70 or 140 mg/kg SBF for 37 d, composited Aβ-induced impairment of memory acquisition, memory retention, and re-learning was reversed, which suggests that SBF has potential value for treatment of AD.

In the present study, light and electron microscopic observation showed that the rats microinjected with composited Aβ displayed dramatic neuropathological changes, including loss of neurons, nuclear pyknosis, neurofibrillary degeneration, neuronophagia, a significant infiltration of inflammatory cells, and disrupted subcellular structures. However, when rats injected with composited Aβ were treated with SBF for 38 d, the neuropathological changes were ameliorated. These results support our previous studies [8, 9, 19–21] and suggest that the effect of SBF on memory deficits induced by composited Aβ may be derived primarily from improving neuron survival.

## Conclusion

In summary, the current findings show that SBF can improve composited Aβ-induced memory deficits and neurodegeneration, which suggests that SBF may be particularly useful in the treatment of neurodegenerative diseases such as AD.

### Additional files

**Additional file 1.** The site of RHTGF-β1 by right intracerebroventricular injection.

**Additional file 2.** The site of Aβ 25-35 and AlCl3 by right intracerebroventricular injection.

## Abbreviations

Aβ: beta-amyloid
AlCl3: aluminum trichloride
RHTGF-β1: recombinant human transforming growth factor-β1
Composited Aβ: amyloid beta protein 25–35 in combination with aluminum trichloride and recombinant human transforming growth factor-β1
AD: Alzheimer’s disease
SP: senile plaques
NFT: intracellular neurofibrillary tangles
SBF: *Scutellaria barbata* flavonoids
SR: screening ratio
HE: hematoxylin-eosin
ANOVA: analysis of variance
PSD: postsynaptic density
