# Supplementary figures and images for: Scutellaria barbata flavonoids alleviate memory deficits and neuronal injuries induced by composited Aβ in rats
Source: Behav Brain Funct. 2016 Dec 8;12:33. doi: 10.1186/s12993-016-0118-8 (PMC5146811; doi:10.1186/s12993-016-0118-8)

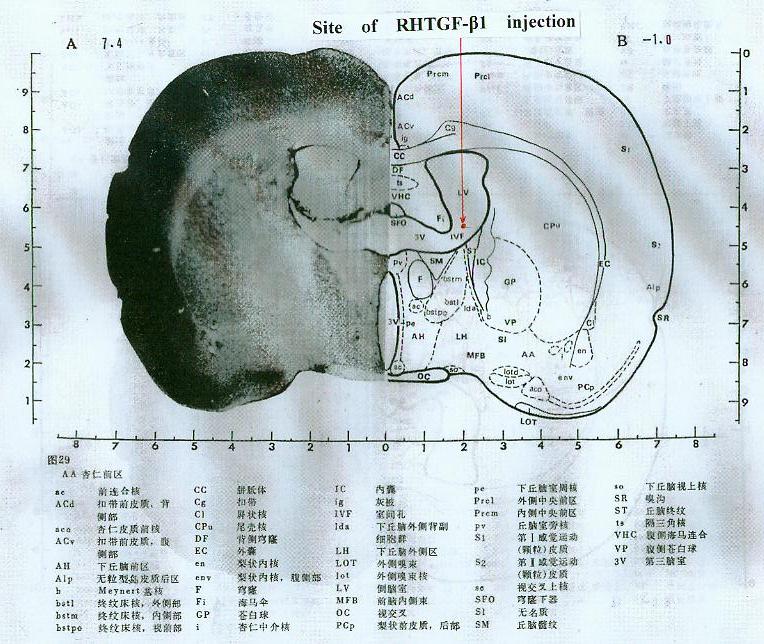

Supplement: Supplementary file 1 — Additional file 1. The site of RHTGF-β1 by right intracerebroventricular injection. [file 12993_2016_118_MOESM1_ESM.jpg]

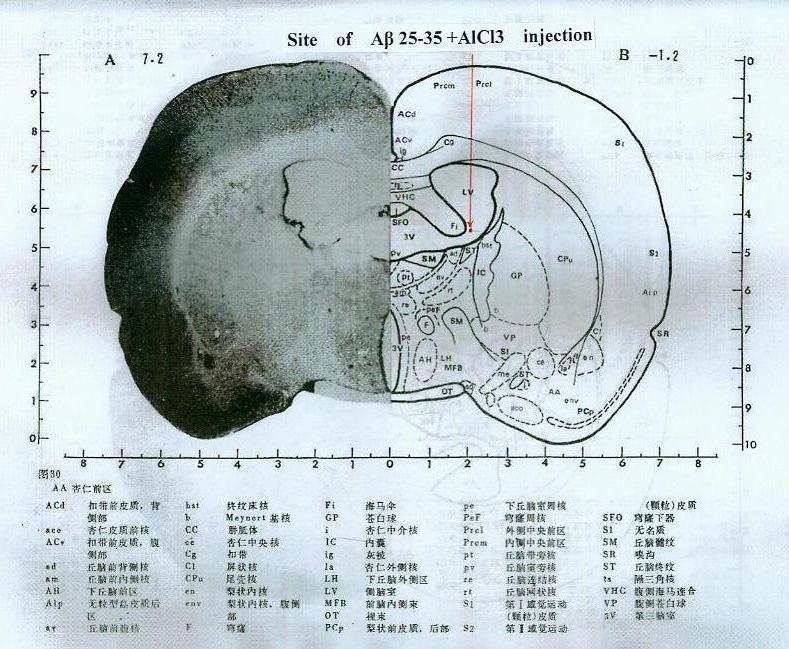

Supplement: Supplementary file 2 — Additional file 2. The site of Aβ 25-35 and AlCl3 by right intracerebroventricular injection. [file 12993_2016_118_MOESM2_ESM.jpg]
